# Influence of the Size of Damage to the Steel Wire Rope on the Magnetic Signature

**DOI:** 10.3390/s22218162

**Published:** 2022-10-25

**Authors:** Paweł Mazurek, Maciej Roskosz, Jerzy Kwaśniewski

**Affiliations:** Department of Machinery Engineering and Transport, Faculty of Mechanical Engineering and Robotics, AGH University of Science and Technology, 30-059 Krakow, Poland

**Keywords:** steel wire ropes, diagnostics, non-destructive testing, passive magnetic method, residual magnetic field

## Abstract

This article presents diagnostic tests of wire ropes using passive magnetic methods. The study used two types of wire ropes with different constructions and diameters. Defects of various depths were modeled in the ropes, which reflected the degree of loss of metallic cross-section. After a series of measurements, a correlation was observed between the amplitude of the module signal and the degree of damage to the rope. The signals were recorded with the advantage of the SpinMeter-3D magnetometer. The obtained results were subjected to the extraction of features, the analysis of which allowed the damage to be identified.

## 1. Introduction

### 1.1. Background

Wilhelm Albert produced the first steel wire rope almost two hundred years ago. It is difficult to imagine a world without steel wire ropes—they are found in almost every industry sector (mining, personal lifts, cableways). For various reasons, defects can inevitably occur in the case of wire ropes, such as wear, wire breakage, strand breakage, rust, and fatigue [1]. Therefore, industry and scientists are investigating various wire rope failure-detection methods to guarantee their safety and reliability [2]. The most commonly used methods are visual and magnetic examination. The easiest way to magnetize steel wire rope is via an electromagnet. However, the change in the magnetization of the rope also occurs due to the work of this rope and the associated magneto-mechanical effects [3]. The sensitivity and high accuracy requirements in the testing inspection of wire rope lead to different sensors and methods. Ref. [4] states that when the defect length is considerable, the fluctuation value of the magnetic flux detection signal caused by the defect is proportional to the area loss rate. Ref. [5] showed that detection results’ accuracy is strictly related to the internal defect depth, geometry size characteristics of the detection instrument, magnetically sensitivity coefficient, lifting-off of the sensor, defect spacing, etc. Magnetic Flux Leakage testing is promising [6,7,8]. Unfortunately, each of the methods and techniques mentioned above has its drawbacks, which outweigh the advantages. Therefore, a new means of wire rope diagnosis is still being sought. The possibility of presenting the estimated mechanical stress while only relying on the Earth’s weak natural magnetic field was proven in [9]. 

### 1.2. Aim of the Work

A novel procedure for diagnosis of steel wire rope with passive magnetic methods is proposed in this work. The changes in the magnetic state of the rope were analyzed. The proposed research method does not require the measurement of the initial state of the rope in the actual location of its operation and periodic diagnostic measurements [10]. The problem of determining the exact technical condition of the tested wire rope depends on many factors. In this article, the authors pay special attention to the influence of the depth of damage of the tested object for the diagnostic magnetic signature. This article examines a new rope and the same rope with an artificially introduced discontinuity (several wires were cut). The authors found no studies on the correlation between the degree of damage to the wire rope and the magnetic signature. This article presents preliminary studies on the impact of changing the rope cross-section for the diagnostic signature. 

## 2. Materials and Methods

### 2.1. Materials

**Figure 1:**
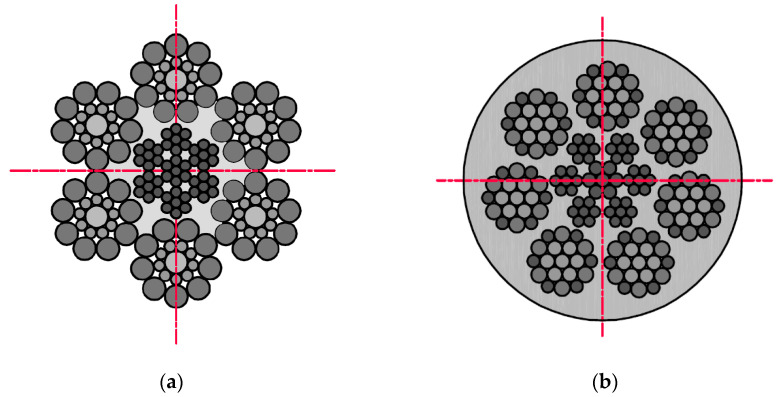
Cross-sections steel wire ropes used in the examination: (**a**) rope no. 1. Ø6.0 mm 6 × 19S + IWRC(7 × 7); (**b**) rope no. 2. Ø6.5 mm 7 × 19W + IWRC(7 × 7)—with polyurethane coating.

The examination was conducted using two different diameters and construction steel wire ropes (Figure 1). Rope no. 1 was a steel wire rope without a polyamide coating, and rope no. 2 was covered with polyurethane material. This solution is used in industry to increase the adhesion of the ropes to the friction wheel. The ropes have different structures and numbers of strands. This combination of ropes was prepared due to the different diameters of the metallic cross-section of individual ropes: no. 1–6 mm, no. 2–5 mm.

**Figure 2:**
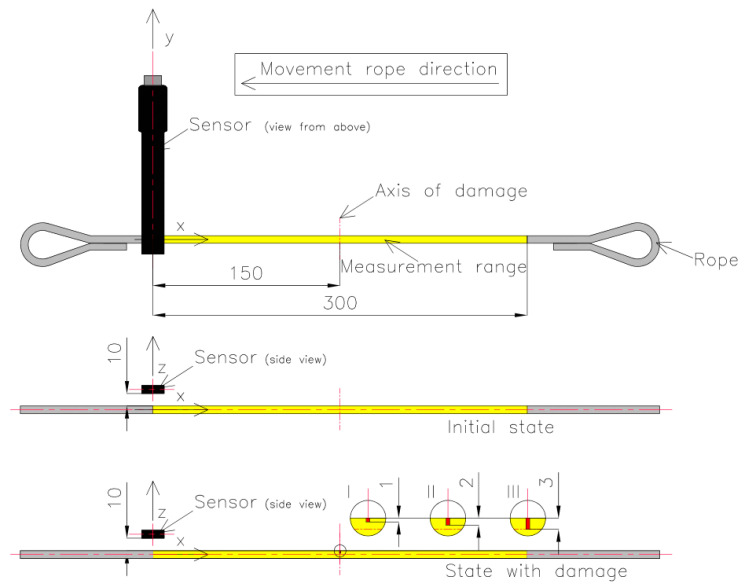
Measurement system: initial (delivery) state and state with damage: 1 mm (I), 2 mm (II), 3 mm (III).

The magnetic sensor was placed on a test strand. Each rope was cut into pieces 700 mm long, and loops were formed at the ends so they could be attached to the test stand. The measuring range of each rope was 300 mm. Each rope realized a reciprocating movement with a speed of approx. 3 mm/s. The measuring range of the steel wire rope was marked with yellow. The room where the measurement was performed did not contain other ferromagnetic elements that could affect the measurement result. The measurement consisted of four stages. In the first part of the measurement, each steel wire rope was installed on the delivery state on the stand. The SpinMeter-3D was positioned 10 mm (in the z-direction) and 0 mm (in the x- and y-directions) from the magnetic sensor (Figure 2). In this condition, in the center of the measured rope, the sensor was calibrated.

**Figure 3:**
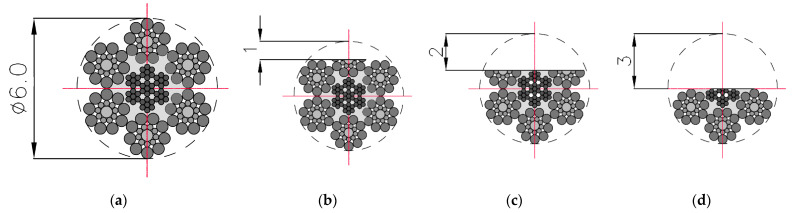
Cross-sections of steel wire rope no. 1 used in examination: (**a**) initial state [P = 100%]; (**b**) damage I—1 mm [P = 93%]; (**c**) damage II—2 mm [P = 71%]; (**d**) damage III—3 mm [P = 50%].

**Figure 4:**
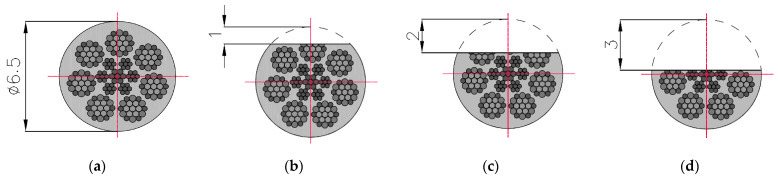
Cross-sections of steel wire rope no. 2 used in examination: (**a**) initial state [P = 100%]; (**b**) damage I—1 mm [P = 96%]; (**c**) damage II—2 mm [P = 75%]; (**d**) damage III—3 mm [P = 56%].

The three components of induction were recorded five times for this state. The mean of all values was used for the final analysis. Then, centrally in the middle of the rope, a discontinuity was introduced (we cut the rope) to a depth of approx. 1 mm and width of approx. 1 mm (I) (Figure 3). The three components of induction were recorded five times for this state. The depth was 2 mm (II) and 3 mm (III) in the following steps, and the width was approx. 1 mm. All recorded signals for the rope having a discontinuity are marked as _D. For later analysis, the metallic rope cross-section factor P was introduced, defined as the ratio of the rope cross-section for a given state to the total metallic cross-section (Figure 4).

### 2.2. Methods

#### 2.2.1. Magnetic Anomaly Detection

By using highly sensitive magnetic sensors, we can capture magnetic anomalies that may result from defects in the tested ropes. The most significant advantage of magnetic anomaly detection is that it is entirely passive compared to the traditional active electromagnetic method, making it possible to develop a low-power security system for magnetic detection.

#### 2.2.2. Self-Magnetic Flux Leakage (SMFL)

Remanent magnetization appears in the rope at the stage of wire production, and its value changes during its stretching and bending. The magnetized ferromagnetic material is therefore considered a magnet. The magnetic field originating from Earth, encountering the gap created by the defect, cannot maintain such a large amount of magnetic field per unit of volume and starts to leak from the material. 

Due to the Earth’s natural magnetic field, it is possible to detect discontinuities using the residual magnetic field of the ferromagnetic ropes. 

The essence of the conducted research is the measurement and interpretation of the local magnetic field disturbance caused by the occurrence of places of stress concentration in the material, local plastic deformation of the material, or the presence of material discontinuities, both mechanical (cracks, delamination) and structural (inclusions of other material). The measured value is the value of the selected magnetic field strength component measured near the object being diagnosed [11,12,13,14].

#### 2.2.3. Tunneling Magnetoresistance (TMR) Sensor-Based Measuring System 

TMR sensors that offer an alternative passive technology are based on the TMR heterostructures, generally comprised of two ferromagnetic phases separated by a thin insulating tunnel barrier called a magnetic tunnel junction (MTJ) [15,16].

For the measurement of the magnetic field, the SpinMeter-3D was used [17]. The SpinMeter-3D uses the phenomenon of tunneling magnetoresistance (TMR). The measuring range of the sensor is ±1000 μT, resolution < 100 nT, and the noise level is 0.25 μT RMS. Figure 3 shows the sensor with the system of measuring axes, which enable measurement of the three components of magnetic field [18].

## 3. Results

**Figure 5:**
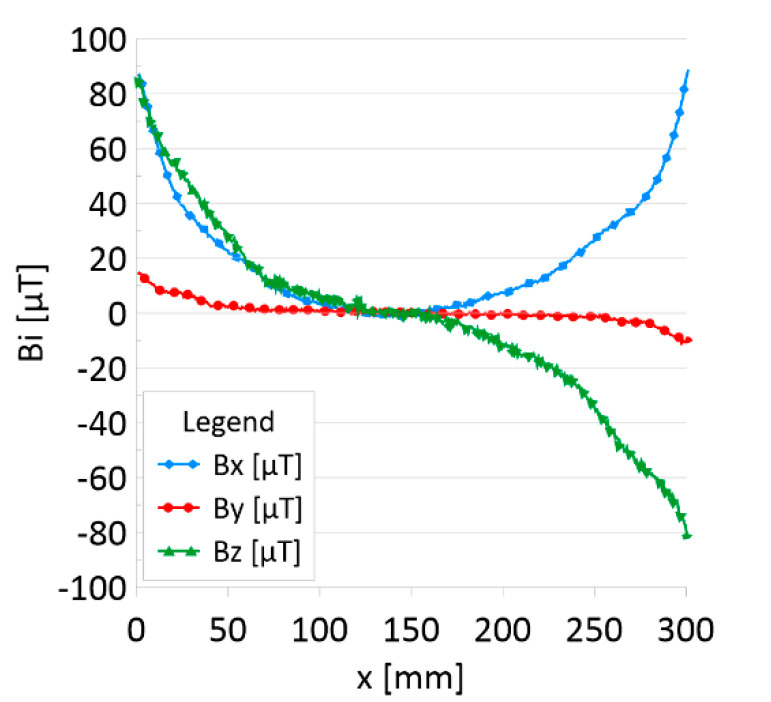
Distribution of the magnetic induction B components along rope no. 1—initial state.

**Figure 6:**
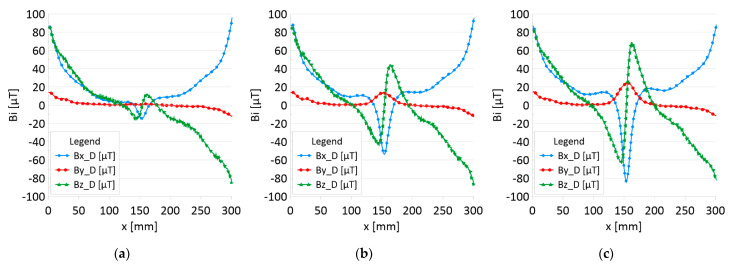
Distribution of the magnetic induction B components along rope no. 1—the state with damage: (**a**) I—1 mm; (**b**) II—2 mm; (**c**) III—3 mm.

**Figure 7:**
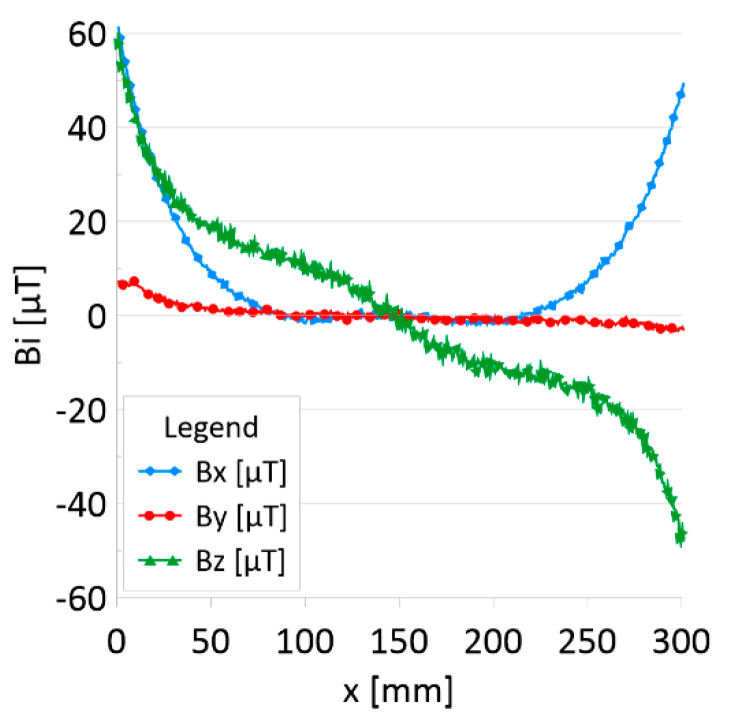
Distribution of the magnetic induction B components along rope no. 2—initial state.

**Figure 8:**
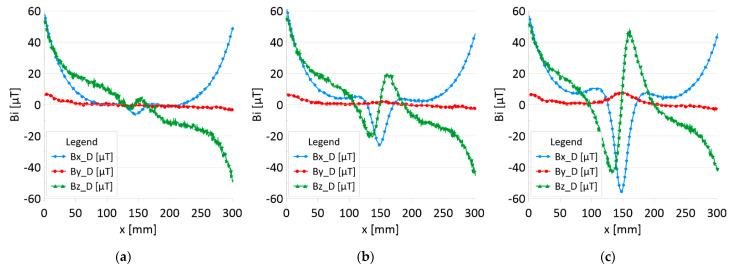
Distribution of the magnetic induction B components along the rope no. 2—state with damage: (**a**) I—1 mm; (**b**) II—2 mm; (**c**) III—3 mm.

**Figure 9:**
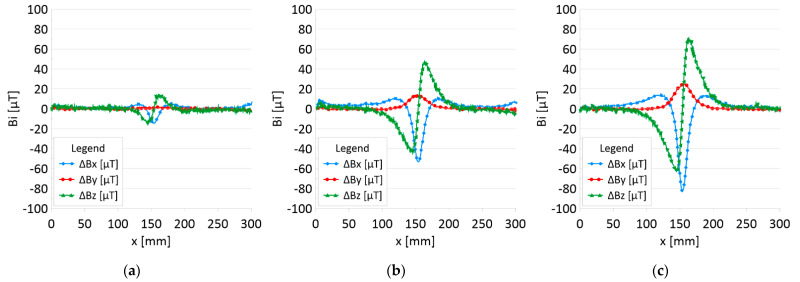
Difference *ΔB* between the magnetic inductions recorded in state with discontinuities: (**a**) I—1 mm; (**b**) II—2 mm; (**c**) III—3 mm and initial state for rope no. 1.

**Figure 10:**
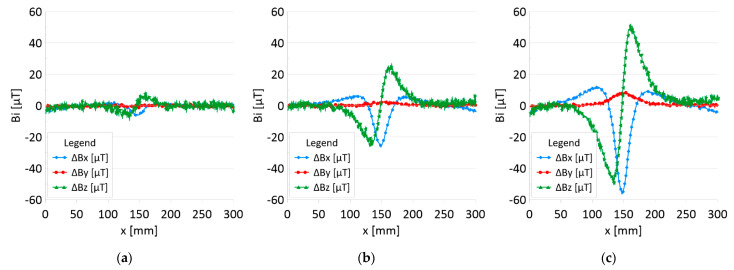
Difference *ΔB* between the magnetic inductions recorded in state with discontinuities: (**a**) I—1 mm; (**b**) II—2 mm; (**c**) III—3 mm and initial state for rope no. 2.

**Figure 11:**
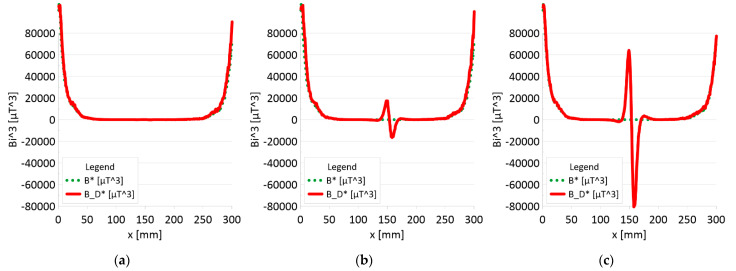
Analysis Bx*By*Bz (B*) and Bx_D*By_D*Bz_D (B_D*) along rope no 1 for discontinuities: (**a**) I—1 mm; (**b**) II—2 mm; (**c**) III—3 mm.

**Figure 12:**
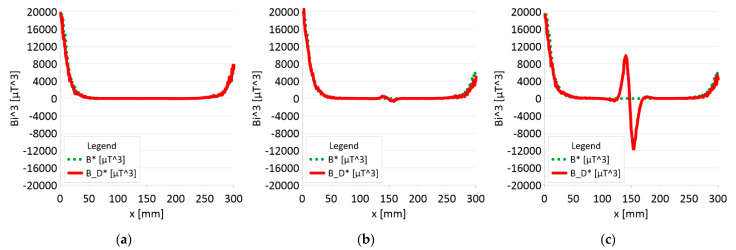
Analysis Bx*By*Bz (B*) and Bx_D*By_D*Bz_D (B_D*) along rope no 2 for discontinuities: (**a**) I—1 mm; (**b**) II—2 mm; (**c**) III—3 mm.

**Figure 13:**
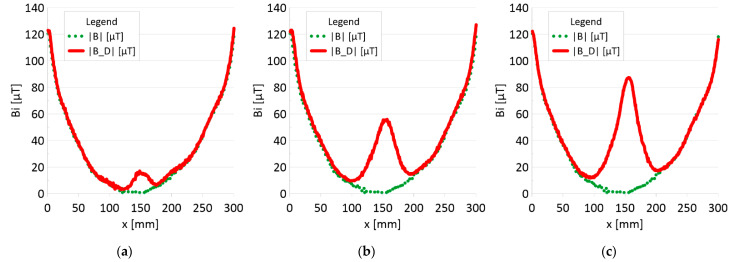
Analysis |B| and |B_D| along rope no 1 for discontinuities: (**a**) I—1 mm; (**b**) II—2 mm; (**c**) III—3 mm.

**Figure 14:**
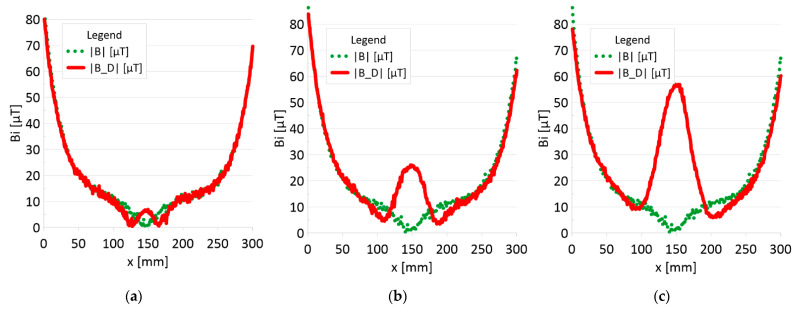
Analysis |B| and |B_D| along rope no 2 for discontinuities: (**a**) I—1 mm; (**b**) II—2 mm; (**c**) III—3 mm.

The results of the obtained measurements are shown in Figure 5, Figure 6, Figure 7, Figure 8, Figure 9, Figure 10, Figure 11, Figure 12, Figure 13 and Figure 14. Figure 5 shows the distribution of the magnetic induction B components along rope no. 1 in the initial state, and Figure 6 shows the signals for rope no. 1 with introduced discontinuities. Figure 7 shows the distribution of the magnetic induction B components along rope no. 2 in the initial state, and Figure 8 shows the signals for rope no. 2 with introduced discontinuities. Figure 9 shows the difference in values between the signal for damaged rope no. 1 and its initial state (ΔB=B_D−B). Figure 10 shows the difference in values between the signal for damaged rope no. 2 and its initial state. Figure 11 illustrates the signal modules calculated as $\left|B\right|=\sqrt{B_{x}*B_{x}+B_{y}*B_{y}+B_{z}*B_{z}}$ for rope no. 1; Figure 12 illustrates this for rope no. 2. In Figure 13, one can observe a new variable not encountered before in diagnostics, determined by the product of signals from all three axes $B^{*}=B_{x}*B_{y}*B_{z}$ for rope no. 1; Figure 14 shows this for rope no. 2.

## 4. Discussion

### 4.1. Interpretation of Charts

Figure 5 and Figure 7 show the diagnostic signal in three directions: x, y, and z, along with the measuring range in the initial state. Figure 5 relates to rope no. 1; Figure 7 to rope no. 2. Figure 6 and Figure 8 show the distribution of the magnetic induction B components along the ropes (Figure 6—rope no. 1; Figure 8—rope no. 2) in the state with three different damages: I—1 mm; II—2 mm; III—3 mm. The difference in the defectograms for almost every axis is apparent. Figure 9 and Figure 10 show the difference in the level of magnetic induction for the initial state of the rope and the state with the modeled damage.

➢ Rope no. 1:
  ○ Damage 1 mm: *ΔBx_D* = 20 μT, *ΔBy_D*= 2 μT*, ΔBz_D* = 30 μT,
  ○ Damage 2 mm: *ΔBx_D* = 60 μT, *ΔBy_D*= 15 μT*, ΔBz_D* = 90 μT,
  ○ Damage 3 mm: *ΔBx_D* = 100 μT, *ΔBy_D*= 25 μT*, ΔBz_D* = 130 μT,
➢ Rope no. 2:
  ○ Damage 1 mm: *ΔBx_D* = 5 μT, *ΔBy_D=1* μT*, ΔBz_D* = 10 μT,
  ○ Damage 2 mm: *ΔBx_D* = 30 μT, *ΔBy_D=5* μT*, ΔBz_D* = 50 μT,
  ○ Damage 3 mm: *ΔBx_D* = 70 μT, *ΔBy_D=10* μT*, ΔBz_D* = 100 μT,

Figure 11 and Figure 12 show extremes in the course of the product of induction components measured on the surface of a defective rope and no extremes in the study of a rope without a defect. For all the ropes, we can see a clear picture of discontinuity.

➢ Rope no. 1:
  ○ Damage 1 mm: B_D* = 1000 μT^3,
  ○ Damage 2 mm: B_D* = 35000 μT^3,
  ○ Damage 3 mm: B_D* = 145000 μT^3,
➢ Rope no. 2:
  ○ Damage 1 mm: B_D* = 50 μT^3,
  ○ Damage 2 mm: B_D* = 1500 μT^3,
  ○ Damage 3 mm: B_D* = 22000 μT^3,

Figure 13 and Figure 14 show the product of three signals and the value of the resultant vector.

➢ Rope no. 1:
  ○ Damage 1 mm: *|B_D|* = 18 μT,
  ○ Damage 2 mm: *|B_D|* = 57 μT,
  ○ Damage 3 mm: *|B_D|* = 89 μT,
➢ Rope no. 2:
  ○ Damage 1 mm: *|B_D|* = 5 μT,
  ○ Damage 2 mm: *|B_D|* = 25 μT,
  ○ Damage 3 mm: *|B_D|* = 57 μT,

### 4.2. Analysis of the Results

Regardless of the initial state of the wire rope magnetism, the damage caused a change in the measured signal. Further research will allow us to determine whether the rope structure could have caused such a result. The signal’s shape resembles a peak for the x-component (tangent along the measurement direction). The y-component (the tangent perpendicular to the measurement direction) is also a peak but with a value several times smaller. Otherwise, the z-component signal (normal) resembles a sine wave shape. Only for the last two measurements (II—2 mm and III—3 mm), the analysis B_D* indicated damage. The excellent diagnostic signal seems to be the product of all three components, which have not been used before. Determining the modulus of signals based on all three components is the most promising. All measurements of this analysis indicated damage. Moreover, the signal amplitude value is proportional to the loss of the metallic rope cross-section.

## 5. Conclusions

1. The phenomenon of tunneling magnetoresistance in diagnosing the technical condition of wire ropes is essential. Passive magnetic diagnostics is much more energy-efficient in comparison to active techniques. It does not require generating an external magnetic field related to energy consumption.

2. The induction component distributions along the rope length were measured with and without a defect. Comparing the obtained results shows that the difference in the defectograms for almost every component is apparent. Even without knowing the initial state, the magnetic anomaly caused by the discontinuity of the material gives a sufficiently strong diagnostic signal to enable its detection.

3. It is necessary to further search for appropriate methods of analyzing the diagnostic signal of steel ropes. If the loss of metal cross-section is less than 10%, the B_D * analysis (product of components) does not show any changes. However, these changes are very clearly visible in the case of the analysis of the |B_D| module.

4. The value of the loss of the metallic section of the wire rope seems to be proportional to the amplitude of the module diagnostic signal from the three signal components.

5. In this article, the authors introduced the product of the magnetic field induction components measured on the rope surface, which was an excellent diagnostic signal, giving a clear image of the discontinuity in the signal distribution.

6. A comprehensive approach using many features of the diagnostic signal is the basis for developing a new passive magnetic method for evaluating the defectoscopic condition of steel ropes.

7. This article does not analyze the influence of the distance between the sensor and the rope—in all cases, it was a constant value of 10 mm. Future research will verify the impact of changing the distance between the tested object and the sensor.

8. In further work, the influence of the structure (the number of strands in the rope) and the diameter on the measurement result will also be analyzed.

## Data Availability

Not applicable.
